# Association between dietary intake and the prevalence of tumourigenic bacteria in the gut microbiota of middle-aged Japanese adults

**DOI:** 10.1038/s41598-020-72245-7

**Published:** 2020-09-16

**Authors:** Daiki Watanabe, Haruka Murakami, Harumi Ohno, Kumpei Tanisawa, Kana Konishi, Yuta Tsunematsu, Michio Sato, Noriyuki Miyoshi, Keiji Wakabayashi, Kenji Watanabe, Motohiko Miyachi

**Affiliations:** 1 Department of Physical Activity Research, National Institutes of Biomedical Innovation, Health and Nutrition (NIBIOHN), 1-23-1 Toyama, Shinjuku-ku, Tokyo, 162-8636 Japan; 2 Department of Pharmaceutical Sciences, University of Shizuoka, 52-1 Yada, Suruga-ku, Shizuoka, 422-8526 Japan; 3 School of Food and Nutritional Sciences, University of Shizuoka, 52-1 Yada, Suruga-ku, Shizuoka, 422-8526 Japan

**Keywords:** Nutrition, Colorectal cancer, Oncogenesis

## Abstract

The relative contribution of diet to colorectal cancer (CRC) incidence is higher than that for other cancers. Animal models have revealed that *Escherichia coli* containing polyketide synthase (*pks*^+^ *E. coli*) in the gut participates in CRC development. The purpose of this cross-sectional study was to examine the relationship between dietary intake and the prevalence of *pks*^+^ *E. coli* isolated from the microbiota in faecal samples of 223 healthy Japanese individuals. Dietary intake was assessed using a previously validated brief-type self-administered diet history questionnaire. The prevalence of *pks*^+^ *E. coli* was evaluated using faecal samples collected from participants and specific primers that detected *pks*^+^ *E. coli*. The prevalence of *pks*^+^ *E. coli* was 26.9%. After adjusting for baseline confounders, the prevalence of *pks*^+^ *E. coli* was negatively associated with the intake of green tea (odds ratio [OR], 0.59 [95% confidence interval (CI) 0.30–0.88] per 100 g/1,000 kcal increment) and manganese (OR, 0.43 [95% CI 0.22–0.85] per 1 mg/1,000 kcal increment) and was positively associated with male sex (OR, 2.27 [95% CI 1.05–4.91]). While futher studies are needed to validate these findings, these results provide insight into potential dietary interventions for the prevention of CRC.

## Introduction

Colorectal cancer (CRC) is the third most common malignant tumour and the second most common cancer-related mortality in the world^1^. According to the temporal profiles and demographic predictions, approximately 2.2 million people will develop CRC, and 1.1 million people will die of the disease by 2030^2^. The incidence and mortality of CRC tend to be higher in high-income countries, suggesting that a Western lifestyle could be a contributing factor^2–4^. Therefore, to reduce the number of CRC patients in the coming decades, it is essential to promote a lifestyle that mitigates CRC risk factors caused by the Western lifestyle typical of high-income countries.

Colibactin is a genotoxic secondary metabolite produced by organisms harbouring the polyketide synthase (*pks*) genomic island, including a certain gut *Escherichia coli* strain with a specific *pks* (*pks*^+^ *E. coli*)^5–7^. Colibactin alkylates the host cell DNA^8^, which leads to genomic instability by generating the DNA inter-strand cross-links that are potentially involved in the development of CRC^8^. Transient infection of mammalian cells with *pks*^+^ *E. coli* leads to cell cycle arrest^5,7^ and DNA double-strand breaks^5,6,9^. Previous studies have reported that the prevalence of *pks*^+^ *E. coli* isolated from the colonic epithelium is higher in CRC patients^10^, patients with familial adenomatous polyposis^11^, and patients with inflammatory bowel disease^10^ compared to healthy individuals. Therefore, *pks*^+^ *E. coli* may be a gut microbiome-related CRC risk factor.

Recently, reports have shown that changes in the faecal microbiome may occur in the very early stages of CRC^12^, leading to an increased risk of disease progression^13,14^. Moreover, among all cancer types, diet reportedly has the highest relative contribution to CRC incidence^15^. In fact, the effect of diet on the onset of CRC is greater than that of smoking or obesity^16^. Poor diet has also recently been associated with potentially adverse gut microbiome profiles in the fecael matter^17^, and colonic mucosa^18^ of healthy individuals. Although a causal relationship between dietary intake and CRC risk through the gut microbiome has not been established, reduced CRC risk by dietary intervention may be the most reasonable, and cost-effective method. Moreover, the relationship between dietary intake and the prevalence of *pks*^+^ *E. coli*, which is, to our knowledge, a risk factor for CRC, has not been elucidated. It is, therefore, essential to clarify this relationship as a means to potentially inform the design of CRC preventative measures. Hence, the purpose of this exploratory study was to examine the relationship between dietary intake and the prevalence of *pks*^+^ *E. coli* isolated from faecal matter in healthy Japanese individuals.

## Results

### Participant characteristics

The characteristics of participants with or without *pks*^+^ *E. coli* in the analysis cohort are presented in Table 1. Of the 223 participants included in the study, 60 were assigned to the *pks*^+^ *E. coli* group (26.9%). The prevalence of *pks*^+^ *E. coli* was significantly higher in males than in females. Age, serum triglyceride levels, and a family history of cancer were slightly lower in the *pks*^+^ *E. coli* group than in the *pks*^*-*^* E. coli* group, however, these differences were not significant. Meanwhile, there were slightly more people who smoked and were employed within the *pks*^+^ *E. coli* group compared to the *pks*^*-*^* E. coli* group, however, the results were not significantly.

**Table 1:** Baseline characteristics of particpants with or without *pks*^+^ *E. coli.*

	*pks*^+^ *E. coli *(*n* = 60)	*pks*^-^ *E. coli *(*n* = 163)	*p* value
Age (years)^1^	57.2 (13.1)	59.1 (12.2)	0.313
Women [*n* (%)]^2^	37 (61.7)	128 (78.5)	**0.011**
BMI (kg/m^2^)^1^	22.4 (2.6)	22.5 (2.8)	0.815
Current smoking [*n* (%)]^2^	4 (6.7)	4 (2.5)	0.251
Hypertension [*n* (%)]^2^	12 (20.0)	27 (16.6)	0.549
Hyperlipidemia [*n* (%)]^2^	12 (20.0)	40 (24.5)	0.477
FH of cancer [*n* (%)]^2^	29 (48.3)	97 (59.5)	0.137
Antibiotics use [*n* (%)]^2^	8 (13.3)	18 (11.2)	0.662
Probiotics use [*n* (%)]^2^	6 (10.0)	22 (13.7)	0.457
Step counts (step/day)^1^	9,611 (2,871)	9,624 (3,390)	0.987
Employment [*n* (%)]^2^	43 (71.7)	97 (59.5)	0.096
Sleep time (min/day)^1^	405 (53)	395 (62)	0.245
Haemoglobin (g/dL)^1^	13.4 (1.3)	13.4 (1.1)	0.778
AST (IU/L)^1^	22.3 (6.4)	23 (7.1)	0.467
ALT (IU/L)^1^	18 (8.5)	18.5 (10.8)	0.757
γ-GTP (IU/L)^1^	28.7 (25.1)	29.6 (29.9)	0.831
FPG (mg/dL)^1^	87.7 (9.3)	86.8 (8.4)	0.487
FSI (µU/mL)^1^	3.6 (1.8)	4.1 (4.5)	0.405
HbA1c (%)^1^	5.5 (0.4)	5.5 (0.3)	0.584
Triglyceride (mg/dL)^1^	81.5 (46.2)	91.5 (61.0)	0.249
HDL-C (mg/dL)^1^	67.7 (21.9)	69.2 (17.3)	0.586
LDL-C (mg/dL)^1^	125 (29)	128 (30)	0.417

The *p* values shown in bold are statistically significant (*p* < 0.05).

*ALT* alanine transaminase, *AST* aspartate transaminase, *BMI* body mass index, *FH* family history, *FPG* fasting plasma glucose, *FSI* fasting serum insulin, *HbA1c* haemoglobin A1c, *HDL-C* high-density lipoprotein-cholesterol, *LDL-C* low-density lipoprotein-cholesterol, *SD* standard deviation, *γ-GTP* γ-glutamyl transpeptidase.

^1^Continuous variables are shown as mean with SD and were analysed by unpaired t-test. BMI was calculated as body weight (kg) divided by height squared (m^2^).

^2^Category variables are shown as the number of individuals (%) and were analysed using a *Χ*^2^ test.

### Intake of food and beverages, and the ***pks***^+^***E. coli*** status

A comparison of food and beverage intake between participants with and without *pks*^+^ *E. coli* is shown in Table 2. The median green tea consumption was significantly lower in the *pks*^+^ *E. coli* group than in the *pks*^-^ *E. coli* group (107.1 g, interquartile range [IQR] 21.4–324.6 vs 150.0 g, IQR 53.6–375.0*, p* = 0.019). In addition, after adjusting for energy intake using the density method, the median green tea consumption was significantly lower in the *pks*^+^ *E. coli* group compared to the *pks*^-^ *E. coli* group (53.6 g/1,000 kcal, IQR 12.4–148.2 vs 86.7 g/1,000 kcal, IQR 29.8–257.7,* p* = 0.010). The same trend was observed in the total amount of non-alcoholic beverages consumed, which included green tea. The median egg intake was significantly lower in the *pks*^+^ *E. coli* group than in the *pks*^-^ *E. coli* group (18.3 g/1,000 kcal, IQR 11.1–29.3 vs 22.8 g/1,000 kcal, IQR 14.0–33.2*, p* = 0.043). No difference was observed in the intake of other diets, beverages, or food groups between the two groups.

**Table 2:** Comparison of crude or energy-adjusted food and beverage intake between participant with and without *pks*^+^ *E. coli.*

	Crude				Energy-adjusted by the density method			
		*pks*^+^ *E. coli *(*n* = 60)	*pks*^-^ *E. coli *(*n* = 163)	*p* value		*pks*^+^ *E. coli *(*n* = 60)	*pks*^-^ *E. coli *(*n* = 163)	*p* value
Cereals	(g/day)	305.1 (185.3–395.1)	290.0 (206.4–358.0)	0.682	(g/1,000 kcal/day)	165.3 (114.8–224.4)	168.4 (133.8–205.7)	0.997
Rice	(g/day)	208.0 (104.0–282.1)	208.0 (117.0–260.0)	0.753	(g/1,000 kcal/day)	115.1 (64.3–159.8)	106.7 (70.1–144.3)	0.726
Noodles	(g/day)	50.4 (33.7–80.8)	48.0 (29.9–80.1)	0.469	(g/1,000 kcal/day)	32.4 (21.6–45.5)	28.8 (19.7–43.9)	0.478
Bread	(g/day)	30.3 (20.0–57.7)	40.0 (20.0–56.0)	0.953	(g/1,000 kcal/day)	17.9 (9.8–31.5)	20.4 (11.7–34.2)	0.619
Pulses	(g/day)	53.3 (37.2–92.3)	56.1 (36.0–91.8)	0.820	(g/1,000 kcal/day)	35.8 (22.6–49.7)	35.2 (21.1–56.3)	0.798
Potatoes	(g/day)	27.7 (18.5–59.4)	50 (20.0–60.0)	0.305	(g/1,000 kcal/day)	17.9 (9.9–34.2)	26.3 (12.6–35.2)	0.101
Sugar and confectioneries	(g/day)	41.2 (25.5–66.4)	44.3 (27.7–66.1)	0.760	(g/1,000 kcal/day)	22.7 (17.0–40.1)	26.7 (16.0–39.9)	0.741
Sugar	(g/day)	3.4 (1.9–5.3)	3.9 (2.2–5.4)	0.330	(g/1,000 kcal/day)	2.1 (1.1–3.3)	2.3 (1.5–3.4)	0.194
Confectioneries	(g/day)	35.0 (23.6–62.9)	39.3 (24.3–60.7)	0.769	(g/1,000 kcal/day)	21.1 (14.2–39.4)	23.3 (13.3–37.0)	0.746
Oil	(g/day)	9.5 (7.2–14.0)	9.6 (6.5–13.4)	0.364	(g/1,000 kcal/day)	6.5 (4.6–7.9)	5.7 (4.4–7.7)	0.421
Fruits	(g/day)	124.4 (54.1–194.6)	139.0 (80.2–200.0)	0.318	(g/1,000 kcal/day)	68.1 (33.0–115.2)	80.8 (48.4–118.5)	0.288
Total vegetables	(g/day)	255.2 (181.2–437.4)	284.1 (208.5–383.6)	0.775	(g/1,000 kcal/day)	163.6 (115.9–234.4)	168.2 (125.2–228.6)	0.430
Green and yellow vegetables	(g/day)	97.7 (54.4–153.7)	103.5 (62.8–152.9)	0.674	(g/1,000 kcal/day)	54.0 (36.9–89.6)	60.0 (38.7–87.3)	0.378
Other vegetables	(g/day)	129.5 (105.0–224.3)	136.4 (97.7–197.1)	0.847	(g/1,000 kcal/day)	82.1 (58.5–114.1)	82.6 (60.5–112.8)	0.875
Pickled vegetables	(g/day)	8.1 (2.8–19.5)	8.2 (2.8–18.1)	0.840	(g/1,000 kcal/day)	4.7 (1.5–10.1)	4.8 (1.5–10.7)	0.674
Mushrooms	(g/day)	11.3 (5.2–18.5)	11.3 (4.5–20.5)	0.924	(g/1,000 kcal/day)	6.8 (3.3–11.4)	6.6 (3.2–12.1)	0.985
Seaweeds	(g/day)	13.2 (4.4–21.6)	11.1 (4.9–17.1)	0.993	(g/1,000 kcal/day)	6.3 (2.3–12.4)	6.9 (3.3–11.2)	0.592
Alcoholic beverages	(g/day)	15.9 (0.0–171.0)	33.9 (3.6–169.6)	0.368	(g/1,000 kcal/day)	9.4 (0.0–80.5)	19.9 (2.0–111.3)	0.341
Non-alcoholic beverages	(g/day)	567.6 (384.8–768.6)	701.2 (477.9–899.0)	**0.029**	(g/1,000 kcal/day)	314.8 (235.1–495.2)	405.4 (294.9 to 520.7)	**0.021**
Fruit and vegetable juice	(g/day)	15.4 (0.0–79.7)	15.4 (0.0–71.4)	0.805	(g/1,000 kcal/day)	11.9 (0.0–39.9)	11.0 (0.0–48.8)	0.925
Green tea	(g/day)	107.1 (21.4–324.6)	150.0 (53.6–375.0)	**0.019**	(g/1,000 kcal/day)	53.6 (12.4–148.2)	86.7 (29.8–257.7)	**0.010**
Black and oolong tea	(g/day)	21.4 (10.0–61.9)	53.6 (10.0–150.0)	0.129	(g/1,000 kcal/day)	13.4 (4.7–42.0)	27.6 (5.1–88.2)	0.100
Coffee	(g/day)	375.0 (107.1–433.1)	173.2 (107.1–375.0)	0.425	(g/1,000 kcal/day)	166.7 (60.0–230.5)	141.9 (69.5–218.7)	0.810
Soft drinks	(g/day)	13.3 (0.0–61.8)	13.3 (0.0–28.6)	0.388	(g/1,000 kcal/day)	7.6 (0.0–31.5)	6.9 (0.0–15.9)	0.481
Fish and shellfish	(g/day)	60.9 (46.6–118.6)	72.6 (48.3–112.1)	0.628	(g/1,000 kcal/day)	41.2 (29.7–64.6)	45.8 (30.8–64.2)	0.516
Meat	(g/day)	71.6 (51.0–95.8)	71.1 (55.5–96.0)	0.965	(g/1,000 kcal/day)	43.7 (32.3–51.6)	42.6 (31.2–55.9)	0.969
Eggs	(g/day)	27.2 (19.4–53.8)	42.4 (23.6–56.6)	0.092	(g/1,000 kcal/day)	18.3 (11.1–29.3)	22.8 (14.0–33.2)	**0.043**
Dairy products	(g/day)	160.3 (67.6–210.4)	160.7 (115.7–220.7)	0.694	(g/1,000 kcal/day)	90.2 (54.1–137.7)	99.0 (69.4–130.9)	0.557

The *p* values shown in bold are statistically significant (*p* < 0.05). All variables are shown as the median with the interquartile range and were analysed using the Mann–Whitney U test.

### Nutrient intake and the prevalence of ***pks***^+^***E. coli***

A comparison of the energy and nutrient intake between participants with or without *pks*^+^ *E. coli* is shown in Table 3. All nutrient values are derived from food, not supplements. There was no significant difference observed in the overall crude mean nutrient intake between the two groups. However, after adjusting for energy intake using the density method, the average intake of riboflavin (mean 0.80 g/1,000 kcal, standard deviation [SD] 0.17 vs mean 0.86 g/1,000 kcal, SD 0.19*, p* = 0.026), folate (mean 210 g/1,000 kcal, SD 69 vs. mean 233 g/1,000 kcal, SD 75*, p* = 0.035), iron (mean 4.7 g/1,000 kcal, SD 1.1 vs. mean 5.0 g/1,000 kcal, SD 1.2*, p* = 0.044), and manganese (mean 1.60 g/1,000 kcal, SD 0.46 vs. mean 1.83 g/1,000 kcal, SD 0.58*, p* = 0.003) was significantly lower in the *pks*^+^ *E. coli* group compared to the *pks*^-^ *E. coli* group. There were no significant differences observed in other nutrient intake between the two groups.

**Table 3:** Comparison of the crude or energy-adjusted energy and nutrient intake between participants with and without *pks*^+^ *E. coli.*

	Crude					Energy-adjusted by the density method			
		*pks*^+^ *E. coli *(*n* = 60)		*pks*^-^ *E. coli *(*n* = 163)	*p* value		*pks*^+^ *E. coli *(*n* = 60)	*pks*^-^ *E. coli *(*n* = 163)	*p* value
Energy	(kcal/day)	1768 (544)		1712 (410)	0.465				
Protein	(g/day)	72.3 (26.9)		72.3 (21.5)	0.982	(% energy/day)	16.4 (3.0)	16.9 (2.9)	0.251
Fat	(g/day)	56.9 (19.4)		55.2 (17.0)	0.557	(% energy/day)	29.1 (4.6)	28.9 (5.1)	0.810
Saturated fat	(g/day)	15.7 (5.8)		15.2 (5.0)	0.521	(% energy/day)	8.07 (1.82)	7.94 (1.76)	0.642
Monounsaturated fat	(g/day)	20.2 (7.2)		19.5 (6.4)	0.510	(% energy/day)	10.31 (1.85)	10.20 (2.02)	0.706
Polyunsaturated fat	(g/day)	13.4 (4.7)		13.1 (4.2)	0.600	(% energy/day)	6.85 (1.25)	6.83 (1.28)	0.946
*n*-6 polyunsaturated fat	(g/day)	10.5 (3.6)		10.2 (3.3)	0.600	(% energy/day)	5.37 (1.02)	5.36 (1.05)	0.930
*n*-3 polyunsaturated fat	(g/day)	2.86 (1.17)		2.78 (1.06)	0.652	(% energy/day)	1.45 (0.38)	1.45 (0.38)	0.992
Marine-origin *n*-3 polyunsaturated fat^1^	(g/day)	1.07 (0.69)		1.06 (0.62)	0.929	(% energy/day)	0.54 (0.28)	0.55 (0.27)	0.738
Eicosapentaenoic acid	(mg/day)	364 (247)		360 (225)	0.920	(% energy/day)	0.18 (0.10)	0.19 (0.10)	0.782
Docosahexaenoic acid	(mg/day)	602 (377)		600 (337)	0.971	(% energy/day)	0.30 (0.15)	0.31 (0.15)	0.664
α-linolenic acid	(mg/day)	1,650 (630)		1,584 (585)	0.482	(% energy/day)	0.84 (0.21)	0.83 (0.20)	0.673
Cholesterol	(mg/day)	395 (198)		412 (153)	0.553	(mg/1,000 kcal/day)	220 (69)	240 (68)	0.060
Carbohydrate	(g/day)	221 (76)		211 (57)	0.368	(% energy/day)	50.0 (7.5)	49.6 (7.4)	0.708
Total dietary fiber	(g/day)	13.0 (5.4)		13.1 (4.3)	0.904	(g/1,000 kcal/day)	7.4 (2.3)	7.8 (2.1)	0.318
Soluble dietary fiber	(g/day)	3.4 (1.5)		3.4 (1.2)	0.805	(g/1,000 kcal/day)	1.9 (0.6)	2.0 (0.6)	0.272
Insoluble dietary fiber	(g/day)	9.2 (3.8)		9.2 (3.0)	0.937	(g/1,000 kcal/day)	5.2 (1.6)	5.5 (1.5)	0.321
Alcohol	(g/day)	9.6 (16.0)		9.6 (15.3)	0.986	(% energy/day)	3.79 (5.86)	3.92 (6.17)	0.886
Retinol	(µg/day)	530 (282)		505 (461)	0.619	(µg/1,000 kcal/day)	308 (160)	290 (272)	0.545
Vitamin A (retinol equivalent)^2^	(µg/day)	897 (397)		890 (524)	0.904	(µg/1,000 kcal/day)	514 (183)	517 (292)	0.913
α-carotene	(µg/day)	487 (325)		496 (346)	0.856	(µg/1,000 kcal/day)	272 (174)	292 (201)	0.460
β-carotene	(µg/day)	3,943 (2,365)		4,139 (2,481)	0.590	(µg/1,000 kcal/day)	2,199 (1,146)	2,441 (1,394)	0.191
β-carotene equivalents^3^	(µg/day)	4,372 (2,564)		4,578 (2,668)	0.599	(µg/1,000 kcal/day)	2,441 (1,243)	2,696 (1,489)	0.201
Cryptoxanthin	(µg/day)	365 (309)		381 (319)	0.749	(µg/1,000 kcal/day)	209 (162)	217 (159)	0.750
α-tocopherol	(mg/day)	8.01 (3.02)		7.99 (2.61)	0.959	(mg/1,000 kcal/day)	4.54 (0.99)	4.66 (1.00)	0.404
Vitamin K	(µg/day)	354 (195)		354 (166)	0.999	(µg/1,000 kcal/day)	199 (91)	209 (90)	0.477
Thiamin	(mg/day)	0.85 (0.31)		0.84 (0.27)	0.967	(mg/1,000 kcal/day)	0.48 (0.10)	0.49 (0.11)	0.342
Riboflavin	(mg/day)	1.41 (0.52)		1.46 (0.44)	0.474	(mg/1,000 kcal/day)	0.80 (0.17)	0.86 (0.19)	**0.026**
Niacin	(mg/day)	18.6 (6.7)		18.8 (6.0)	0.888	(mg/1,000 kcal/day)	10.7 (2.6)	11.0 (2.4)	0.475
Vitamin B_6_	(mg/day)	1.37 (0.51)		1.39 (0.43)	0.789	(mg/1,000 kcal/day)	0.78 (0.19)	0.82 (0.17)	0.223
Vitamin B_12_	(µg/day)	10.35 (5.85)		10.47 (5.42)	0.889	(µg/1,000 kcal/day)	5.78 (2.51)	6.05 (2.61)	0.486
Folate	(µg/day)	369 (159)		394 (145)	0.296	(µg/1,000 kcal/day)	210 (69)	233 (75)	**0.035**
Pantothenic acid	(mg/day)	6.94 (2.46)		6.94 (1.97)	0.995	(mg/1,000 kcal/day)	3.94 (0.73)	4.06 (0.70)	0.267
Vitamin C	(mg/day)	126 (67)		138 (58)	0.258	(mg/1,000 kcal/day)	73 (32)	81 (31)	0.064
Sodium	(mg/day)	4,137 (1,366)		4,030 (1,140)	0.591	(mg/1,000 kcal/day)	2,370 (462)	2,367 (433)	0.967
Potassium	(mg/day)	2,847 (1,068)		2,905 (921)	0.709	(mg/1,000 kcal/day)	1,628 (407)	1,707 (390)	0.196
Calcium	(mg/day)	603 (275)		627 (241)	0.553	(mg/1,000 kcal/day)	340 (108)	367 (112)	0.109
Magnesium	(mg/day)	270 (94)		274 (80)	0.751	(mg/1,000 kcal/day)	154 (31)	161 (30)	0.136
Phosphorus	(mg/day)	1,117 (432)		1,126 (344)	0.879	(mg/1,000 kcal/day)	631 (133)	658 (124)	0.179
Iron	(mg/day)	8.22 (3.17)		8.51 (2.70)	0.541	(mg/1,000 kcal/day)	4.7 (1.1)	5.0 (1.2)	**0.044**
Zinc	(mg/day)	8.33 (2.94)		8.18 (2.27)	0.727	(mg/1,000 kcal/day)	4.7 (0.6)	4.8 (0.8)	0.343
Copper	(mg/day)	1.13 (0.40)		1.12 (0.30)	0.864	(mg/1,000 kcal/day)	0.64 (0.11)	0.66 (0.11)	0.275
Manganese	(mg/day)	2.80 (1.05)		3.05 (0.98)	0.114	(mg/1,000 kcal/day)	1.60 (0.46)	1.83 (0.58)	**0.003**

The *p* values shown in bold are statistically significant (*p* < 0.05). All variables are shown as the mean with standard deviation and were analysed using an unpaired t-test. ^1^Sum of eicosapentaenoic acid, docosapentaenoic acid, and docosahexaenoic acid.

^2^Sum of retinol, β-carotene/12, α-carotene/24, and cryptoxanthin/24.

^3^Sum of β-carotene, α-carotene/2, and cryptoxanthin/2.

### Multivariate analysis of dietary intake and the prevalence of ***pks***^+^***E. coli***

Participant characteristics and dietary intake that were significantly associated with the prevalence of *pks*^+^ *E. coli* were applied to a multivariate analysis (Table 4). The multivariate analysis models, even after adjusting for covariates, showed that: (1) the prevalence of *pks*^+^ *E. coli* was significantly higher in males than in females (odds ratio [OR], OR, 2.27 [95% confidence interval (CI) 1.05–4.91]*, p* = 0.038); and (2) the OR of *pks*^+^ *E. coli* for a 100 g/1,000 kcal increment in green tea consumption per day was 0.59 [95% CI 0.30–0.88*, p* = 0.003]. Regarding nutrients, the OR of *pks*^+^ *E. coli* for a 1 mg/1,000 kcal increment in manganese intake per day was 0.43 [95% CI 0.22–0.85*, p* = 0.012].

**Table 4:** Odds ratios of *pks*^+^ *E. coli* for the intake of energy-adjusted food, beverage, and nutrients calculated by multivariate logistic regression analysis.

	Multivariate adjusted model	
	OR (95% CI)	*p* value
Age (1 years increment)	0.99 (0.97–1.01)	0.411
Male sex^1^	2.27 (1.05–4.91)	**0.038**
BMI (1 kg/m^2^ increment)	0.97 (0.86–1.09)	0.600
Current smoker^2^	3.29 (0.73–14.85)	0.311
FH of cancer^3^	0.63 (0.34–1.17)	0.140
Energy intake (100 kcal/day increment)	1.01 (0.94–1.08)	0.807
Step counts (1,000 steps/day increment)	0.97 (0.87–1.08)	0.621
Green tea (100 g/1,000 kcal/day increment)	0.59 (0.30–0.88)	**0.003**
Eggs (10 g/1,000 kcal/day increment)	0.79 (0.55–1.04)	0.090
Riboflavin (1 mg/1,000 kcal/day increment)	0.29 (0.05–1.86)	0.186
Folate (10 mg/1,000 kcal/day increment)	0.97 (0.92–1.01)	0.148
Iron (1 mg/1,000 kcal/day increment)	0.82 (0.60–1.11)	0.184
Manganese (1 mg/1,000 kcal/day increment)	0.43 (0.22–0.85)	**0.012**

Results are shown as odds ratios (ORs) and 95% confidence intervals (CI). Statistical analysis was carried out using the likelihood ratio test for multivariate logistic analysis, and the ORs and 95% CI were estimated. Bold *p* values are statistically significant (*p* < 0.05). Adjusted factors included age (continuous), sex (female or male), BMI (continuous), smoking status (never smoker, past smoker, or current smoker), family history (FH) of cancer (yes or no), energy intake (continuous), and step counts (continuous).

^1^Reference group: women.

^2^Reference group: never smoker.

^3^Reference group: no FM of cancer.

**Figure 1:**
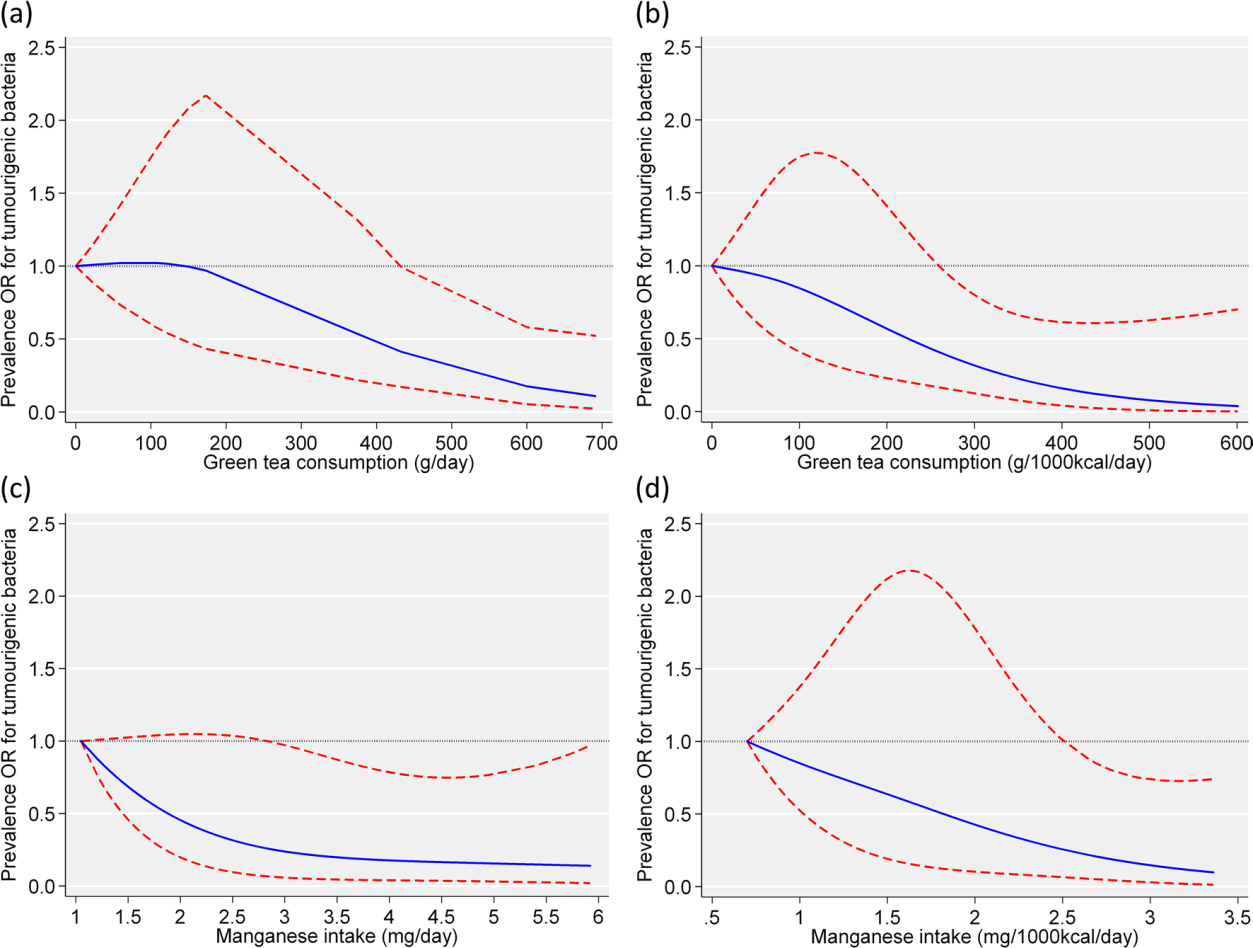
Association of the intake of green tea (**a**,**b**) and manganese (**c**,**d**) with the prevalence odds ratio (OR) of colibactin-producing *E. coli* in a restricted cubic spline logistic regression model. Adjusted factors included age (continuous), sex (female or male), BMI (continuous), smoking status (never smoker, past smoker, or current smoker), family history of cancer (yes or no), energy intake (continuous), and step counts (continuous). The solid line represents the OR. The broken lines show the 95% Confidence Intervals (CIs). If the 95% CI for the OR did not include 1.00, the *p* value was estimated to be < 0.05. If the 95% CI included 1.00, the *p* value was estimated to be ≥ 0.05.

Dietary intake variables that showed a significant association with the prevalence of *pks*^+^ *E. coli* in the multivariate analysis were used in a restricted cubic spline model to evaluate dose-dependent responses to *pks*^+^ *E. coli* (Fig. 1). The analysis of the curves for the dietary intake variables and the prevalence OR of *pks*^+^ *E. coli* showed that: (1) the prevalence OR of *pks*^+^ *E. coli* was significantly lower when green tea consumption exceeded approximately 430 g/day, or 260 g/1,000 kcal/day (reference: those without green tea consumption), and (2) the prevalence OR of *pks*^+^ *E. coli* was significantly lower when the intake of manganese exceeded approximately 2.86 mg/day (reference: 1.05 mg /day), or 2.65 mg/1,000 kcal/day (reference: 0.70 mg/1,000 kcal/day).

## Discussion

The purpose of this cross-sectional study was to examine the relationship between dietary intake and the prevalence of *pks*^+^ *E. coli* in healthy Japanese individuals. After adjusting for confounding factors, our results showed a significant negative association between the prevalence of *pks*^+^ *E. coli* and the intake of green tea and manganese. In addition, we showed a significantly higher prevalence of *pks*^+^ *E. coli* in males than in females. To our knowledge, this is the first study to show a significant association between dietary intake and the prevalence of *pks*^+^ *E. coli*. Furthermore, the results of this study support a hypothesis suggesting an association between dietary intake and CRC risk.

Unlike other cancers, such as lung cancer, no single risk factor accounts for most cases of CRC. Apart from age and male sex, the following risk factors for CRC incidence have been identified and established in previous epidemiological studies: family history of cancer, inflammatory bowel disease, smoking, obesity, diabetes, excessive alcohol consumption, and high consumption of red and processed meat^3^. These established CRC risk factors have been associated with potentially adverse gut microbiome profiles^13,14^, indicating the importance of evaluating the prevalence of tumourigenic bacteria in the gut microbiota. This study indicated that the prevalence of *pks*^+^ *E. coli* isolated from faecal matter was 26.9% in our cohort. According to previous studies, the prevalence of *pks*^+^ *E. coli* isolated from the colonic epithelium was 20.8% in healthy UK^10^ and 22.0% in healthy US individuals^11^. The samples used to evaluate the prevalence of *pks*^+^ *E. coli* in previous studies and this current study were tissue and faecal matter, respectively. Although previous studies, as well as the current one, have different evaluation methods for the prevalence of *pks*^+^ *E. coli*, the results are relatively similar. Moreover, our results indicating that the prevalence of *pks*^+^ *E. coli* was significantly higher in males than in females supports those of previous studies that have reported the risk of CRC to be higher in males compared to females^3,19^. In other words, our results on the prevalence of *pks*^+^ *E. coli* are similar to those from previous studies in high-income countries, showing that participant characteristics in our study were unbiased and appropriate.

Taken together the key findings of the current study are as follows: (1) a negative association exists between green tea consumption, and manganese intake and the prevalence of *pks*^+^ *E. coli*; (2) a significantly lower OR of *pks*^+^ *E. coli* was observed in individuals with a daily intake of 430 g/day green tea compared to those without. Note, the manganese intake derived from green tea contributed to 75.6% of total manganese intake in this cohort, and most of the inter-individual variance in manganese intake was attributed to green tea consumption. Hence, the negative association observed between the intake of manganese and the prevalence of *pks*^+^ *E. coli* may be contributory to that of green tea consumption as the results lost significance following adjustment for green tea consumption. The accuracy of estimation of green tea intake by a brief-type self-administered diet history questionnaire (BDHQ) showed a moderate correlation with green tea intake estimated by dietary records (male, *r* = 0.68; female, *r* = 0.64). However, it has been reported that the accuracy of the estimated median green tea intake is overestimated by 20–23%^20^. Therefore, a minimum effective amount of green tea, which was significantly associated with reduced prevalence of *pks*^+^ *E. coli* in a dose–response relationship, may be 20% lower than approximately 430 g/day (i.e. ~ 340 g [2–3 cups/day]).

To our knowledge, there are two systematic reviews^21,22^, five prospective cohort studies^23–27^, and four case and control studies^28–31^ that have evaluated the relationship between green tea consumption and CRC risk; however, no consensus has been reached regarding the anti-CRC properties in these studies. The two systematic reviews concluded that the anti-CRC effects of green tea are inadequate and contradictory^21,22^. Only studies targeting East Asians have reported the anti-CRC effects of green tea^26,27,29–31^. Meanwhile, low green tea consumption in non-Asian countries may contribute to the non-significant results of these studies^28^. Green tea consumption for 7 days exhibited beneficial effects in improving lymphocytic DNA damage in middle-aged healthy non-smokers^32^. In addition, green tea catechins have anti-inflammatory properties^33,34^, which help to mitigate against oxidative tissue injury^34^. Subsequent changes in the gut microbiota and reduced intestinal inflammation may then be related to the anti-inflammatory properties of green tea and green tea polyphenols^35^. Although there are no studies describing the relationship between green tea consumption and the prevalence of *pks*^+^ *E. coli*, one study has described the effects of green tea consumption on the gut microbiome. Yuan et al. performed an intervention study in healthy Chinese adults and found a significant increase in the Firmicutes to Bacteroidetes ratio isolated from faecal matter after 2 weeks of green tea consumption (400 mL/day)^36^. Firmicutes and Bacteroidetes are two major bacterial phyla that dominate the human gut microbiota. The Firmicutes to Bacteroidetes ratio increases from birth to adulthood and is decreased in advanced ages^37^. Interestingly, higher frequencies of colibactin-producing *E. coli* and enterotoxigenic *Bacteroides fragilis* in the colonic epithelium were observed in patients with familial adenomatous polyposis compared to healthy individuals^11^. Their study also reported that mice with guts co-colonised with colibactin-producing *E. coli* and *B. fragilis* had a higher tumour growth rate due to increased levels of interleukin-17 in the colon, as well as DNA damage in the colonic epithelium compared to mice with either bacterial strain alone^11^. These results suggest that green tea consumption significantly reduces the prevalence of *pks*^+^ *E. coli* by suppressing the growth of certain microorganisms in the gut microbiome. The detailed mechanisms and causal relationships must be clarified with further intervention studies and fundamental studies.

The strength of this study is the verified association between the prevalence of *pks*^+^ *E. coli* and dietary intake estimated using a validated dietary assessment tool. By using the approach described above, this study generated a new hypothesis for the association between diet and the prevalence of *pks*^+^ *E. coli* as a tumourigenic bacteria. However, this study has certain methodological limitations. First, the temporal and direct causal relationship observed between dietary intake and the prevalence of *pks*^+^ *E. coli* could not be inferred as this study is a cross-sectional study. Second, although our results show that green tea and manganese intake is negatively associated with the prevalence of *pks*^+^ *E. coli*, these food and nutrient intakes estimated by BDHQ have not been fully validated against objective biomarkers. The results may have been affected by systematic errors due to body mass index and gender^38^. In addition, when we examined the association between diet and the prevalence of *pks*^+^ *E. coli*, we focused exclusively on diet estimated from a validated BDHQ. We have previously reported that yoghurt consumption increases stool frequency^39^. The results suggest that yoghurt consumption may affect the gut microbiome. Therefore, it is necessary to verify our results using a validated dietary assessment tool other than the BDHQ. Further, we conducted an exploratory investigation of the association between the intake of specitic foods, beverages, and nutrients and the prevalence of *pks*^+^ *E. coli*; however, multiple testing problems may arise when multiple tests are used to calculate *p* values. Finally, there is the possibility of selection bias due to higher health awareness of the participants in this study than in the general population. Of the 750 participants in the Nutrition and Exercise Intervention Study (NEXIS) cohort, 259 adults agreed to participate. As the participation rate is relatively low, volunteer bias may occur. In addition, participants are all from Tokyo metropolitan area with an average age of approximately 58 years. These limitations may prevent the generalisation of the results. Therefore, future studies with larger randomised samples should be used to investigate further the association between diet and the prevalence of *pks*^+^ *E. coli*. In addition, the effects of green tea consumption and the intake of manganese on the risk of *pks*^+^ *E. coli* should be examined by prospective cohort studies and randomised intervention studies.

Given the rapid Westernisation of diet around the world, there is an urgent need to highlight the importance of diet in the prevention of CRC. Furthermore, the difference in dietary intake between groups may explain the large global differences in cancer burden^40^. Therefore, to develop sustainable, comprehensive, and effective public health programmes for CRC prevention, our study data will provide useful insights into the development effective preventative intervention strategies for CRC.

## Methods

### Participants and study procedure

Of the 750 participants in the NEXIS cohort (ethical approval number: kenei102; clinical trial registration number: NCT00926744), which is operated and managed by the National Institute of Health and Nutrition, NIBIOHN since 2012, 259 adult males and females from the Tokyo metropolitan area, Japan, agreed to participate in this study (ethical approval number: kenei 3-04; clinical trial registration number: UMIN000023270). The health-related variables (e.g. smoking status, family history of disease, drugs use, and dietary survey) survey and faecal sampling in this study were conducted between September 2015 and December 2017.

The questionnaire for the lifestyle survey and a kit for faecal collection and storage were mailed to the participants. We used a triaxial accelerometer (Actimarker; EW4800; Panasonic Co., Ltd, Japan) to measure daily step counts as an objective index of physical activity. The participants were instructed to complete the questionnaire for the lifestyle survey (e.g. dietary survey) and collect lumps of faeces of ~ 2 cm diameter (approximately 3 g) at home. The collected faeces were immediately placed in a sealed container and stored in a − 20 °C freezer. The participants were instructed to bring the questionnaire and faecal samples to the National Institute of Health and Nutrition, NIBIOHN within 5 days after faecal collection, at which point they received health examinations, such as anthropometric and blood tests. The investigators, qualified as registered dieticians or nurses, checked the questionnaires and interviewed those with unanswered questions or unclear responses to confirm their answers. A portion of the frozen faeces was transported to the University of Shizuoka by a refrigerated truck and tested for the presence of *pks*^+^ *E. coli*. Blood samples were used to measure conventional risk factors for lifestyle-related diseases, such as haemoglobin A1c, triglyceride, and low-density lipoprotein-cholesterol. The study protocol was reviewed and approved by the Research Ethical Review Committee of NIBIOHN (approval number: kenei102 and kenei 3-04). Study procedures, as well as the risks associated with participation, were explained and written informed consent was obtained from all participants. Moreover, all study procedures were performed in accordance with relevant standard international guidelines/regulations.

Of the participants included in the baseline analysis (*n* = 259) who provided informed consent, those with a history of cancer (*n* = 12), gastrointestinal disease (*n* = 3), diabetes mellitus (*n* = 13), renal failure (*n* = 1), and cardiovascular disease (*n* = 6) were excluded from subsequent analyses. In addition, a participant with extremely low/high mean energy intake estimated by the BDHQ (*n* = 1) was excluded from the analysis as an outlier (< 600 or > 4,000 kcal/day)^41^. As a result, 223 participants were ultimately included in this study.

### Confirmation of ***pks***^+^***E. coli*** using polymerase chain reaction (PCR)

To confirm that the *E. coli* was a *pks*^+^ strain, PCR was performed to amplify the genes from the *clb* cluster using the bacterial genomic DNA as a template. The details have been reported elsewhere^42^. In brief, the following 16 primer sets were used for amplifying each of the genes in the cluster: the primer sets were clbA-F/clbA-R for *clbA*, clbB-F/clbB-R for *clbB*, clbC-F/clbC-R for *clbC*, clbD-F/clbD-R for *clbD*, clbF-F/clbF-R for *clbF*, clbG-F/clbG-R for *clbG*, clbH-F/clbH-R for *clbH*, clbI-F/clbI-R for *clbI*, clbJ-F/clbJ-R for *clbJ*, clbK-F/clbK-R for *clbK*, clbL-F/clbL-R for *clbL*, clbM-F/clbM-R for *clbM*, clbN-F/clbN-R for *clbN*, clbO-F/clbO-R for *clbO*, clbP-F/clbP-R for *clbP*, and clbQ-F/clbQ-R for *clbQ* (see Supplementary Table S1 for primer sequence information). Of these primers, participants for whom *clbB*, *clbJ*, and *clbQ* were detected from faeces were defined as *pks*^+^ *E*. *coli* individual.

### Dietary assessment

Dietary intake was evaluated using the BDHQ of Sasaki et al., consisting of 58 food and beverage items validated against 16-day dietary records (4 days per season)^20,43^. Food and beverage items listed in the BDHQ consist of food and beverages commonly consumed in Japan, according to the national health and nutrition survey^44^. This study examined only the frequency of intake of the 58 food and beverage items in the past month. Participants with unanswered questions or unclear responses were asked to confirm their responses during a face-to-face interview. Dietary and nutrient intakes were calculated from the weight of food intake (i.e. calculated according to the portion size and frequency of dietary intake) and nutritional information listed in the Standard Tables of Food Composition in Japan^45^.

### Statistical analysis

All data were compared between participants with or without the *pks*^+^ *E. coli*. Categorical variables were expressed as numbers and percentages. The chi-square test was used to compare variables between the two groups. Descriptive statistics for continuous variables were expressed as mean and SD or median and IQR, and differences in continuous variables between the two groups were evaluated using the unpaired *t* test or the Mann–Whitney U test. Forty-two nutrient items^43^ and 28 items of food, beverages, and food groups^20^ calculated from the BDHQ were used in this study. The details of the food groups are described elsewhere^20^. In accordance with studies on the validation against the BDHQ, food and beverage consumption was expressed as median and IQR^20^, whereas nutrient intake was expressed as mean and SD^43^ and we conducted the descriptive statistics in accordance with these studies. In this study, to adjust for energy intake, food and nutrient intakes per 1,000 kcal were calculated using the density method^46^. Crude values and energy-adjusted values calculated using the density method for all food and nutrient intake variables were compared between with-without the *pks*^+^ *E. coli*.

Multivariate logistic regression analysis was used to adjust for potential confounding factors related to food and nutrient intake and the prevalence of *pks*^+^ *E. coli*. We adjusted for age (continuous), sex (female or male), body mass index (continuous), smoking status (never smoker, past smoker, or current smoker), family history of cancer (yes or no), energy intake (continuous), and step counts (continuous). These variables were decided with reference to covariates used in previous studies that examined the association between CRC and green tea consumption^23–31^. Participant characteristics, as well as food and nutrient intake that were significantly associated with the prevalence of *pks*^+^ *E. coli* (*p* < 0.05) were used in the analysis. The results of the analysis were expressed as OR and 95% CI. The OR and 95% CI were calculated for food and nutrient intake per unit increment. Food and nutrient intake variables that showed a significant association with *pks*^+^ *E. coli* prevalence in the multivariate analysis were used in a restricted cubic spline model, with three knots placed at the 5th, 50th, and 95th percentiles to evaluate dose-dependent responses to *pks*^+^ *E. coli*^47^. If the 95% CI for the OR did not include 1.00, the *p* value was estimated to be < 0.05. If the 95% CI included 1.00, the *p* value was estimated to be ≥ 0.05.

*p* < 0.05 (two-tailed) was considered statistically significant. All analyses were performed using Stata/MP 15.0 Statistical Software (StataCorp, College Station, TX, USA).

## Supplementary information

Supplementary Information
