# Supplementary material for: Association between dietary intake and the prevalence of tumourigenic bacteria in the gut microbiota of middle-aged Japanese adults
Source: Sci Rep. 2020 Sep 16;10:15221. doi: 10.1038/s41598-020-72245-7 (PMC7495490; doi:10.1038/s41598-020-72245-7)
Supplement: Supplementary file 1 — Supplementary Information [file 41598_2020_72245_MOESM1_ESM.docx]

**Association between dietary intake and the prevalence of tumourigenic bacteria in the gut microbiota of middle-aged Japanese adults**

Daiki Watanabe^1^, Haruka Murakami^1^, Harumi Ohno^1^, Kumpei Tanisawa^1^, Kana Konishi^1^, Yuta Tsunematsu^2^, Michio Sato^2^, Noriyuki Miyoshi^3^, Keiji Wakabayashi^3^, Kenji Watanabe^2^, and Motohiko Miyachi^1*^

1. Department of Physical Activity Research, National Institutes of Biomedical Innovation, Health and Nutrition (NIBIOHN), Tokyo, 162-8636, Japan; d2watanabe@nibiohn.go.jp (D.W.); haruka-m@nibiohn.go.jp (H.M.); ohno.h322@gmail.com (H.O.); tanisawa@waseda.jp (K.T.); konishi@toyo.jp (K.K.); miyachi@nibiohn.go.jp (M.M.)
2. Department of Pharmaceutical Sciences, University of Shizuoka, Shizuoka, 422-8526, Japan; tsunematsu@u-shizuoka-ken.ac.jp (Y.T.); michio@u-shizuoka-ken.ac.jp (M.S.); kenji55@u-shizuoka-ken.ac.jp (Kenji Watanabe)
3. School of Food and Nutritional Sciences, University of Shizuoka, Shizuoka, 422-8526, Japan; miyoshin@u-shizuoka-ken.ac.jp (N.M.); kwakabayashi@u-shizuoka-ken.ac.jp (Keiji Wakabayashi)

*Corresponding author: Motohiko Miyachi, Department of Physical Activity Research, National Institutes of Biomedical Innovation, Health and Nutrition (NIBIOHN), 1-23-1 Toyama, Shinjuku-ku, Tokyo 162-8636, Japan. Phone: ^+^81-3- 3203-8061, E-mail: miyachi@nibiohn.go.jp

Supplementary Information:

**Supplementary Table 1:** Oligonucleotide primer sequences.

**Supplementary Table 1.** Oligonucleotide primer sequences.

| Primer name Sequence | |
| --- | --- |
| clbA-F  clbA-R  clbB-F  clbB-R  clbC-F  clbC-R  clbD-F  clbD-R  clbF-F  clbF-R  clbG-F  clbG-R  clbH-F  clbH-R  clbI-F  clbI-R  clbJ-F  clbJ-R  clbK-F  clbK-R  clbL-F  clbL-R  clbM-F  clbM-R  clbN-F  clbN-R  clbO-F  clbO-R  clbP-F  clbP-R  clbQ-F  clbQ-R  clbP-F  clbP-R | 5'-tttaggggtgatgagtggagaggct-3'  5'-tcatcaaaccagtagagataacttccttcact-3'  5'-tgttccgttttgtgtggtttcagcg-3'  5'-gtgcgctgaccattgaagatttccg-3'  5'-ttgacggaggcgttcgatacttcac-3'  5'-acttgtatcactcggcggcaatcaa-3'  5'-cggagaatgtagtcggcgtccattt-3'  5'-ccctgatttcacgcccaaataccct-3'  5'-cgattgccctcacagagccgaatat-3'  5'-aatgccatgagaaaataaccgccgc-3'  5'-cgaatatgctgcgctgacctgtagt-3'  5'-gatagcgattctccagcagcaggtt-3'  5'-ctttgtcgagttgccggaataccct-3'  5'-tgtgtctgatctcctgtggtccctt-3'  5'-ttgagaatgtacgactgaacccgcc-3'  5'-aatgaatgtccgccagcttcgaaga-3'  5'-tggcctgtattgaaagagcaccgtt-3'  5'-aatgggaacggttgatgacgatgct-3'  5'-ttgatgatcaccacgccagcttctt-3'  5'-gcggatggcggtagtgataagctag-3'  5'-cacaggtgtctatgcccatcgttgt-3'  5'-gccgaccactgagtttgactgctat-3'  5'-tgtttcaaggcgcgggtaagatcat-3'  5'-tagtcactcacggcaacaacacgag-3'  5'-ggcattcagttcgggtatgtgtgga-3'  5'-aacagagctgccgtaaagactcgac-3'  5'-aaggaggtgcggtaaataacgacgg-3'  5'-cggtggcatggatccttttcgtttg-3'  5'-cttgccgcagacaatcgtatcctct-3'  5'-cctggagatagtatacccggtgcga-3'  5'-ctgtgtcttacgatggtggatgccg-3'  5'-gcattaccagattgtcagcatcgcc-3'  5'-cttgccgcagacaatcgtatcctct-3'  5'-cctggagatagtatacccggtgcga-3' |

DNA primers were designed on the basis of sequence data obtained from the *E. coli* genome sequence database.
